# Glucose Metabolism Reprogramming of Regulatory T Cells in Concanavalin A-Induced Hepatitis

**DOI:** 10.3389/fphar.2021.726128

**Published:** 2021-08-31

**Authors:** Chen Huang, Yi Shen, Mengyi Shen, Xiaoli Fan, Ruoting Men, Tinghong Ye, Li Yang

**Affiliations:** ^1^ Department of Gastroenterology and Hepatology, Sichuan University-University of Oxford Huaxi Joint Centre for Gastrointestinal Cancer, Frontiers Science Center for Disease-Related Molecular Network, West China Hospital, Sichuan University, Chengdu, China; ^2^ Laboratory of Liver Surgery, State Key Laboratory of Biotherapy/Collaborative Innovation Center for Biotherapy, West China Hospital, Sichuan University, Chengdu, China

**Keywords:** autoimmune hepatitis, regulatory T cells, glycolysis, oxidative phosphorylation, glucose metabolism reprogramming

## Abstract

Autoimmune hepatitis (AIH) is an inflammatory liver disease caused by a dysregulated immune response. Although the pathogenesis of AIH remains unclear, impaired regulatory T cells (Tregs) have been considered a driver of AIH development. Unlike autoreactive T cells, Tregs mainly utilize oxidative phosphorylation (OXPHOS) as their energy supply. Elevated glycolysis has been reported to limit the suppressive functions of Tregs. However, whether glucose metabolism reprogramming in Tregs is involved in AIH etiology remains unknown. The aim of this study was to examine alternations in Treg numbers and functions in AIH patients and concanavalin A (Con A)-induced hepatitis, while exploring associations between impaired Tregs and glucose metabolism. The frequency of Tregs was decreased in the peripheral blood but increased in liver biopsies of AIH patients. Moreover, immunosuppressive therapy rescued circulating Tregs in AIH. In Con A-induced immune hepatitis, enhanced intrahepatic Treg accumulation was observed over time, accompanied by reduced splenic Treg numbers. To investigate whether functional impairment of Tregs occurs in AIH, Tregs were isolated from experimental AIH (EAH) model mice and normal controls and the former displayed downregulated mRNA levels of FOXP3, CTLA4, CD103, TIGIT, CD39, and CD73. EAH model-derived Tregs also produced fewer anti-inflammatory mediators (TGF-β and IL-35) than control Tregs. Moreover, enhanced glycolysis and reduced OXPHOS were found in Tregs from EAH model mice, as reflected by elevated levels of key glycolytic enzymes (HK2, PK-M2, and LDH-A) and a decreased ATP concentration. This study revealed a decreased peripheral Treg frequency and abnormal intrahepatic Treg infiltration in AIH. It is first reported that glucose metabolism reprogramming is associated with decreases and functional impairments in the Treg population, promoting AIH development. Targeting glucose metabolism may provide novel insights for the treatment of AIH.

## Introduction

Autoimmune hepatitis (AIH) is a relatively rare chronic liver disease mediated by immunological imbalance and characterized by elevated IgG levels, interface hepatitis, and specific autoantibodies (Mieli-Vergani et al., 2018; Trivedi et al., 2019). Prednisolone in combination with azathioprine is widely accepted as a standard therapy to achieve biochemical remission (Lohse et al., 2020; Mack et al., 2020). Although the underlying mechanism is obscure, the disruption of immune homeostasis is commonly considered a major driver of pathogenesis in AIH, which triggers aberrant immune attack on hepatocytes (Floreani et al., 2018; Webb et al., 2018).

Different murine models have been applied to investigate the molecular mechanisms of AIH and evaluate drugs, such as models induced by surrogates (e.g., concanavalin A, Con A; S-100) and autoantigens (e.g., CYP2D6, FTCD), as well as transgenic models (e.g., NTxPD-1^−/−^, APS-1) (Christen, 2019). However, these models have different limitations. The Con A-induced model shows acute and severe liver injury rather than chronic injury (Gantner et al., 1995); the CYP2D6 model has a chronic course but mainly mimics type 2 AIH (Liu et al., 2020); and the NTxPD-1^−/−^ model only represents fetal AIH (Kido et al., 2008). To date, there is no perfect animal model to fully represent the features of AIH patients. Hepatic cellular infiltration in AIH patients shows the predominance of T lymphocytes (Webb et al., 2018). Additionally, abnormal antigen-specific T cell activation is considered a key point in AIH development (Floreani et al., 2018; Mack et al., 2020). Therefore, although Con A-induced hepatitis is acute and self-limited, it is widely used due to the induction of a T cell-mediated immune response in the liver, which can partially mimic the pathogenesis of human AIH (Tiegs et al., 1992; Christen, 2019; Fan et al., 2020; Tan et al., 2020).

As key regulators to maintain peripheral tolerance, regulatory T cells (Tregs) can secrete anti-inflammatory factors and granzymes to suppress autoreactive T cells (Wing et al., 2019). Additionally, dendritic cells are also inhibited by Tregs *via* inhibitory surface receptors. Therefore, Treg deficiency usually contributes to a series of autoimmune diseases, such as systemic lupus erythematosus, rheumatoid arthritis, and inflammatory bowel disease (Xie et al., 2019; Santinon et al., 2020; Quandt et al., 2021). Additionally, impairments in the Treg number and functions in AIH patients have been reported in several studies (Longhi et al., 2004; Longhi et al., 2006; Liberal et al., 2015). However, some studies have shown that Tregs retain their suppressive capacity and are not reduced in number in AIH patients (Peiseler et al., 2012). Reports on Treg changes in AIH patients remain controversial.

It is well known that cellular metabolism is essential for the activation, maintenance, and function of immune cells (Buck et al., 2017; Shyer et al., 2020). Naïve T cells are metabolically inactive and tend to use oxidative phosphorylation (OXPHOS) as their source of energy. In contrast, effector T cells (Teffs) change their energy supply from OXPHOS to glycolysis during activation (Leone and Powell, 2020). Metabolic reprogramming is required to support the proliferation and inflammatory functions of Teffs. Conversely, Tregs rely more on OXPHOS and fatty acid oxidation than glycolysis (Michalek et al., 2011). As an important regulator of metabolic programs, mTOR was reported to inhibit glycolysis in Tregs, leading to enhanced immunosuppressive functions (Charbonnier et al., 2019). Moreover, another study revealed that Ndfip1 could ameliorate autoinflammatory disease by suppressing glycolysis in Tregs (Layman et al., 2017). According to the information above, glycolysis appears to impair the stability and suppressive capacity of Tregs, thereby leading to autoimmune disorders. However, the effect of Treg metabolic reprogramming on the pathogenesis of AIH remains unclear.

The present study explored alterations in the numbers of circulating and intrahepatic Tregs in both AIH patients and Con A-induced model mice. Furthermore, the suppressive capability and glucose metabolism of Tregs from experimental AIH (EAH) model mice were evaluated. Collectively, the findings demonstrated that numerically and functionally impaired Tregs were involved in Con A-induced immune hepatitis *via* glucose metabolism reprogramming.

## Materials and Methods

### Patients

Patients with AIH were recruited at the Division of Gastroenterology and Hepatology, West China Hospital of Sichuan University (Sichuan, China) from October 2019 until May 2021. All patients fulfilled the diagnostic criteria defined by the International Autoimmune Hepatitis Group (Alvarez et al., 1999; Hennes et al., 2008). Peripheral blood samples were drawn from newly diagnosed AIH patients (AIH, *n* = 30), patients given glucocorticoid treatment at different periods (3 weeks, *n* = 15; 6 months, *n* = 18), patients in complete biochemical remission (CR, *n* = 16), and healthy controls (HCs, *n* = 33). Liver biopsy specimens were collected from AIH patients. In addition, the normal liver tissues were obtained from patients undergoing liver resection for benign focal lesions (e.g., hepatic hemangioma and hepatic cysts). All normal liver samples were acquired from the biobank, West China Hospital, Sichuan University. Clinical characteristics are displayed in Table 1. Ethical approval was obtained from the Ethics Committee of West China Hospital, Sichuan University (No. 2013221).

**TABLE 1:** Clinical and biochemical characteristics of AIH patients. Data are presented as the mean ± SD or *n* (%).

	Prior to treatment (*n* = 30)	Under therapy		
		3 weeks (*n* = 15)	6 months (*n* = 18)	Biochemical remission (*n* = 16)
Age (years)	49.6 ± 13.7	50.9 ± 14.6	49.8 ± 7.0	61.0 ± 12.4
Female (n, %)	24 (80.0%)	11 (73.3%)	17 (94.4%)	14 (87.5%)
Cirrhosis (n, %)	10 (33.3%)	4 (26.7%)	2 (11.1%)	7 (43.8%)
Laboratory tests				
Total bilirubin (μmol/L)	94.3 ± 139.3	29.9 ± 16.9	19.3 ± 11.9	14.9 ± 4.8
ALT (IU/L)	181.8 ± 175.9	89.3 ± 55.4	38.3 ± 16.7	20.8 ± 6.1
AST (IU/L)	201.2 ± 203.7	65.5 ± 30.1	47.2 ± 18.9	25.6 ± 4.9
IgG (g/L)	24.0 ± 8.3	18.8 ± 4.1	16.0 ± 2.7	13.5 ± 2.3
ANA positive (*n*, %)	22 (73.3%)	9 (60.0%)	10 (55.6%)	8 (50.0%)

### Cell Isolation

Peripheral blood mononuclear cells (PBMCs) were isolated from whole blood using density gradient centrifugation. Briefly, PBS-diluted blood samples (1:1) were carefully layered on 2 ml human lymphocyte separation medium (Dakewe, Shenzhen, China). Single-cell suspensions were harvested by gradient centrifugation at 800 *g* for 30 min. PBMCs were washed twice for further experiments.

### Animals

Female C57BL/6 mice (8–10 weeks; 20–22 g) were provided by the Experimental Animal Center of Sichuan University (Sichuan, China). Animal experiments were approved by the Animal Ethics Committee of the West China Hospital, Sichuan University (No. 2020388A). EAH was established according to the protocol reported in a previous study (Fan et al., 2020). Mice were randomly divided into a negative control (NC) group and a Con A group. Con A (Sigma-Aldrich, St. Louis, MO, United States ) was administered intravenously *via* tail vein injection at a dose of 10 mg/kg. The mice in the NC group were injected with an equal volume of sterile saline. The mice were sacrificed at 12, 24, 48, or 72 h after injection for sample collection.

### Flow Cytometry Analysis

PBMCs were isolated from AIH patients and healthy volunteers as mentioned above. In addition, single-cell suspensions were obtained by mechanical disruption of mouse spleens through 70-μm cell strainers. Erythrocytes were lysed using lysis buffer. Then, the freshly collected cells were resuspended in 100 μl of PBS and incubated with anti-CD3, anti-CD4, and anti-CD8a antibodies (BioLegend, San Diego, CA, United States ) at 4°C for 30 min. To detect Treg subpopulations, intracellular staining was performed using a transcription factor buffer set (BioLegend, San Diego, CA, United States ). After staining with anti-CD4 and anti-CD25 antibodies, the cells were fixed, permeabilized, and incubated with an anti-FOXP3 antibody (BioLegend, San Diego, CA, United States ). The frequencies of CD4+ CD25+ FOXP3+ Tregs, CD3+ CD4+ T cells, and CD3+ CD8a+ T cells were examined using a flow cytometer (Beckman Coulter, Brea, CA, United States).

### Histopathological Analysis

Liver tissues were preserved in 4% formalin and embedded in paraffin. The samples were sliced into 3–4-μm sections, followed by dewaxing and rehydration. Then, hematoxylin and eosin (H&E) staining was performed according to standard protocols (Feldman and Wolfe, 2014). The pathological changes were evaluated by microscopic examination.

### Immunohistochemical Staining of Liver Specimens

After dewaxing and rehydration, liver sections were immersed in EDTA antigen repair buffer and heated for 15 min in a microwave oven. Endogenous peroxidase activity was blocked with 3% hydrogen peroxide. The slides were blocked with 10% goat serum for 30 min and incubated with a primary antibody against FOXP3 (1:100, Cat #14-5773-82, eBioscience, San Diego, CA, United States ) overnight at 4°C. The sections were washed with PBS and incubated with horseradish peroxidase-conjugated goat anti-rat IgG (1:200, GB23302, Servicebio, Wuhan, China) for 1 h at 37°C. Then, diaminobenzidine solution was added for color development. The staining was visualized by light microscopy.

### Enzyme-Linked Immunosorbent Assay

Serum from patients and HCs was prepared by centrifugation of whole blood at 1,000 g for 10 min. The levels of IL-6, IL-10, and TGF-β were measured using a human ELISA detection kit according to the manufacturer’s instructions (MultiSciences, Hangzhou, China). Briefly, 100 μl of standards, blank, or serum samples were added to the ELISA plate. Then, 50 μl of the assay diluents were pipetted into the wells and incubated for 2 h with shaking. After washing completely, horseradish peroxidase-conjugated streptavidin was added, followed by incubation for 45 min at room temperature. Finally, a tetramethylbenzidine substrate was applied for color development. The optical density values were measured at 450 and 570 nm by a microplate reader (BioTek, Winooski, VT, United States).

### Liver Function Assay

Blood was collected from mice in each group by retro-orbital removal and then centrifuged at 1,000 g for 10 min to obtain the serum. The serum levels of alanine transaminase (ALT) and aspartate transaminase (AST) were detected by an automatic biochemical analyzer (Hitachi, Tokyo, Japan).

### Isolation of CD4 + CD25 + Cells

Splenic mononuclear cells isolated from mice in different groups were separated as described above. First, cells were incubated with antibodies and microbeads at 4°C; then, they were passed through a long deletion column. CD4+ cells were negatively selected with a magnetic-activated cell sorting system (Miltenyi Biotec, Gladbach, Germany). Next, CD4+ CD25+ cells were enriched by positive selection. After magnetic sorting, the purities of CD4+ CD25+ cells and CD4+ CD25+ FOXP3+ Tregs were assessed with a flow cytometer (Beckman Coulter, Brea, CA, United States). Sorted cells were rinsed and collected for further experiments.

### Real-Time Quantitative PCR

Total RNA was extracted from Tregs isolated from mouse spleens with an AxyPrep total RNA extraction kit (Axygen, New York, NY, United States). cDNA was synthesized using a PrimeScrip RT reagent kit (Takara, Shiga, Japan). All primers were obtained from Tsingke (Beijing, China), and the sequences are listed in Supplementary Table S1. Then, RT-qPCR was performed using SYBR Green Supermix on a CFX96 RT-qPCR detection system (BioRad, Hercules, CA, United States). The expression levels of mRNA transcripts related to Treg functions and glucose metabolism were normalized to β-actin mRNA levels.

### ATP Assay

ATP levels in Tregs isolated from mouse spleens were measured with the ATP Assay Kit (Beyotime Biotech, Shanghai, China). A total of 2 × 10^5^ cells were sorted and lysed with ATP assay lysis buffer, followed by centrifugation at 12,000 *g* for 5 min at 4°C. The supernatants were collected and immediately placed on ice. Then, 100 μl of ATP detection working solution was added to each well and incubated at room temperature for 5 min to eliminate any background signal. The sample wells were treated with 20 μl of supernatant. The cellular ATP concentration was measured by a luminometer (PerkinElmer, Waltham, MA, United States).

### Statistical Analysis

Data are presented as the mean ± SD and were analyzed with GraphPad Prims 9 (GraphPad Software Inc., San Diego, CA, United States) and SPSS 24.0 software (SPSS Inc., Chicago, IL, United States). Statistical analysis was performed using a two-tailed Student’s t-test and one-way ANOVA. Statistical significance was labeled according to the *p* value as *p value < 0.05, **p value < 0.01, and ***p value < 0.001.

## Results

### The Frequency of Tregs is Decreased in the Peripheral Blood but Elevated in the Liver of AIH Patients

**FIGURE 1:**
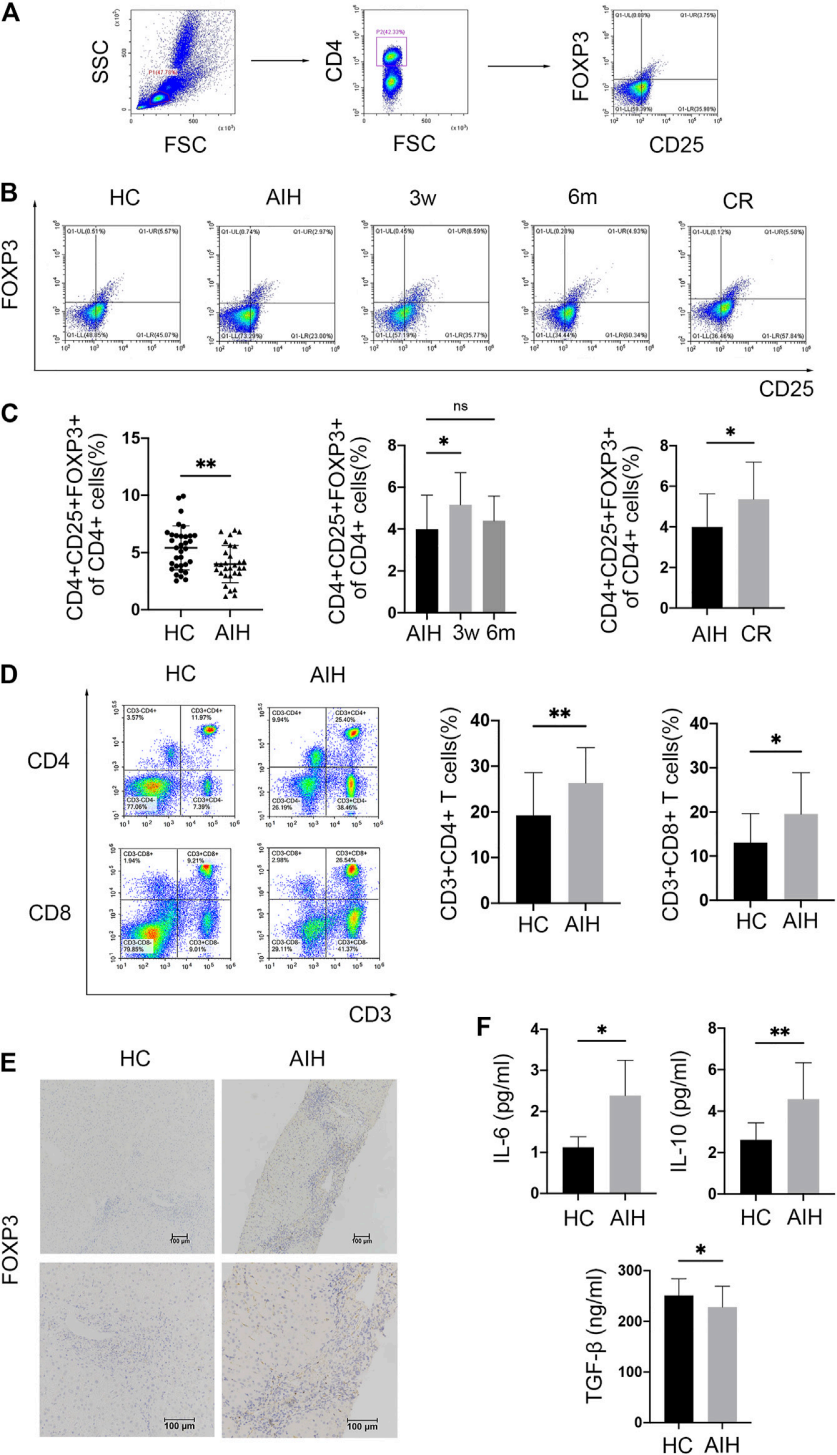
Tregs were decreased in the peripheral blood but accumulated in the liver of AIH patients. **(A)** Gating strategy for circulating CD4+ CD25+ FOXP3+ Tregs. **(B)** Representative FCM plots showing Tregs in healthy donors (HCs), newly diagnosed AIH patients (AIH), patients receiving therapy (3 weeks and 6 months), and patients in complete biochemical remission (CR). **(C)** FCM analysis of circulating Tregs in HCs and AIH patients at diagnosis, during therapy, and in CR. **(D)** FCM analysis of circulating CD3+ CD4+ T cells and CD3+ CD8+ T cells in HCs and AIH patients. **(E)** Immunohistochemical staining for FOXP3 in liver samples from HCs and AIH patients. **(F)** Serum levels of IL-6 and TGF-β measured by ELISA. Original magnification, 100x and 200x.

Tregs were defined as CD4 + CD25 + FOXP3+ cells by flow cytometry (FCM). The gating strategy is provided in Figure 1A. Circulating Tregs in AIH patients and HCs were analyzed by FCM. The results showed that the frequency of Tregs among CD4 + T cells was reduced in the patients compared with the HCs (Figures 1B,C). To determine the effect of glucocorticoid therapy on Treg numbers, the posttreatment FCM data were compared with baseline data. Treg subsets were slightly but significantly elevated after 3 weeks of prednisolone treatment compared with the baseline levels. However, the Treg frequency returned to approximately the baseline level after 6 months of immunosuppressive therapy. Moreover, patients in complete biochemical remission showed enhanced levels of circulating Tregs compared with those measured at the initial diagnosis (Figure 1C). Considering the essential role of T cell-mediated immune attack, the frequencies of T cells were also analyzed in some patients. Activated CD3 + CD4 + T cell levels in AIH patients showed almost 1.4-fold upregulation relative to those in HCs. Similarly, the CD3 + CD8a + T cell frequency in AIH patients was higher than that in HCs (Figure 1D). To further localize Tregs in the liver, liver samples from newly diagnosed patients and HCs underwent IHC staining for FOXP3. FOXP3+ Tregs were strikingly increased and mainly accumulated in portal areas in AIH patients compared with HCs (Figure 1E). To verify the involvement of Treg-related cytokines, human serum levels of TGF-β and IL-10 were detected by ELISA. Cytokine analyses demonstrated that AIH patients had lower TGF-β serum levels than HCs. Nevertheless, the circulating IL-10 level was increased in AIH (Figure 1F). Overall, Tregs were reduced in the peripheral blood but increased in the liver of AIH patients and secreted anti-inflammatory factors.

### Enhanced Intrahepatic Treg Infiltration in EAH Over Time

Given the changes in Treg numbers observed in AIH patients, further experiments were carried out with EAH model mice to explore the potential mechanism.

**FIGURE 2:**
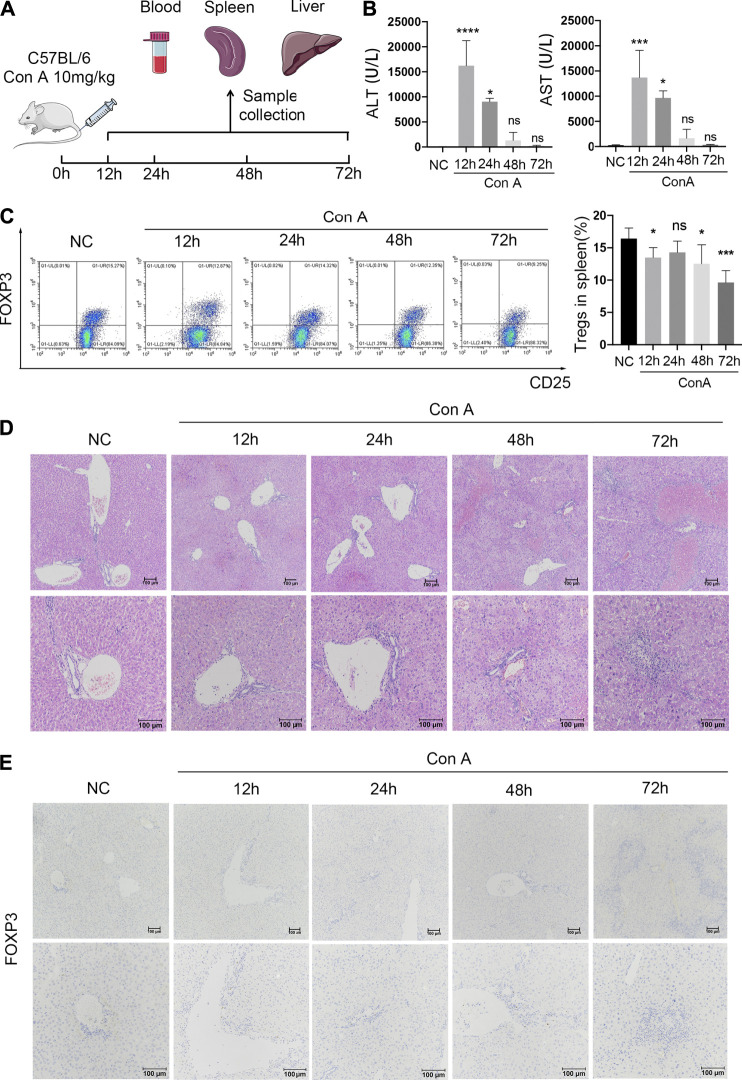
Reduced splenic Tregs and accumulated intrahepatic Tregs in Con A-induced immune hepatitis over time. **(A)** C57BL/6 mice were injected intravenously with 20 mg/kg Con A. Eyeball blood, spleens, and livers were collected separately at 12, 24, 48, and 72 h after Con A treatment. **(B)** Serum ALT and AST levels. **(C)** FCM analysis of splenic Tregs with comparisons between NCs and EAH model mice at different time points. **(D)** H&E staining of murine liver samples. **(E)** IHC staining for FOXP3 to evaluate intrahepatic Treg infiltration in different groups. Original magnification, 100x and 200x.

Mice were sacrificed at 12, 24, 48, or 72 h after Con A injection (Figure 2A). The liver, spleen, and blood were collected. The serum ALT and AST levels in Con A-treated mice were dramatically elevated at 12 h compared with those in NCs and then rapidly decreased between 24 and 72 h after Con A administration (Figure 2B). Representative H&E staining of the liver displayed moderate inflammatory cell infiltration in portal areas and hepatocyte necrosis at 12 h. Moreover, aggravated immune cell infiltration and vacuolation were observed between 24 and 48 h. At 72 h after modeling, livers showed a disorganized hepatic lobular structure, severe sinusoidal congestion, and massive areas of hepatocyte necrosis surrounded by inflammatory cell infiltrates (Figure 2C).

To evaluate alterations in Treg numbers, FCM and IHC analyses were conducted in each group. The FCM data revealed that the frequency of splenic Tregs was decreased at 12 h in the Con A group compared with the normal group. After a slight upregulation at 24 h, the peripheral Treg frequency gradually decreased until 72 h (Figure 2D). In addition, intrahepatic Tregs were identified by IHC staining for FOXP3. In contrast to the splenic Treg data, an increasing number of Tregs infiltrated portal areas over time after modeling (Figure 2E). The results indicate that intrahepatic Treg accumulation increases over time in EAH, which is contrary to the trends for the splenic Treg frequency.

### Treg Inhibitory Function is Impaired in EAH

**FIGURE 3:**
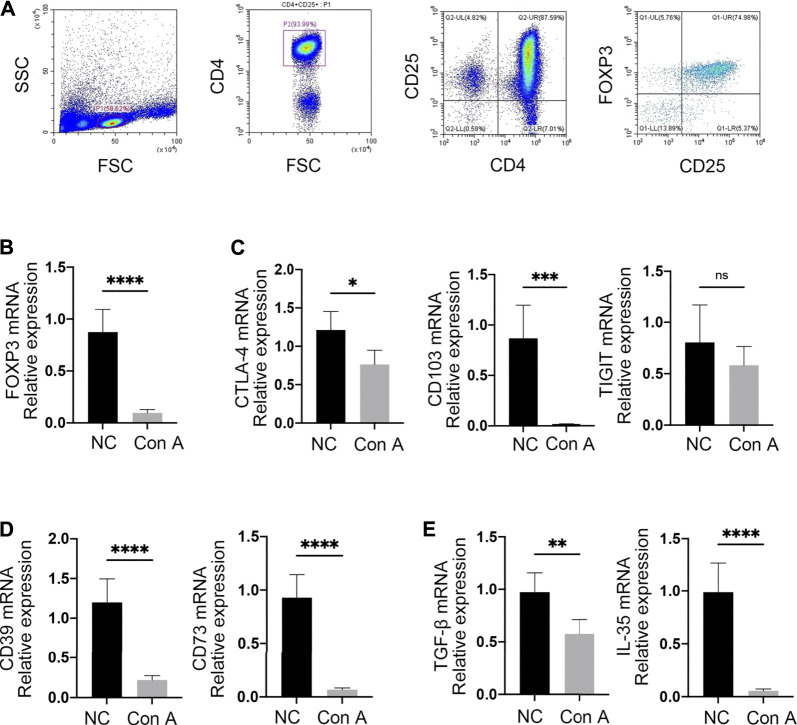
Tregs were functionally impaired in EAH. **(A)** CD4+ CD25+ Tregs were isolated from mouse spleens by immunomagnetic bead sorting. The purities of CD4+ CD25+ and CD4+ CD25+ FOXP3+ cells were assessed by FCM. **(B)** The expression of FOXP3 was examined by RT-qPCR. **(C)** mRNA expression levels of CTLA-4, CD103, and TIGIT. **(D)** mRNA levels of CD39 and CD73 in Tregs from each group. **(E)** Expression of the anti-inflammatory cytokines TGF-β and IL-35. Gene expression was normalized to that of β-actin.

To better explore the suppressive function of Tregs, CD4+ CD25+ cells were isolated from mouse spleens in both the NC and Con A groups by immunomagnetic cell sorting. FCM data showed that the percentage of CD4 + CD25 + cells reached approximately 88%. Moreover, the purity of CD4+ CD25+ FOXP3+ Tregs was approximately 75% (Figure 3A).

To identify whether Tregs exhibit an impaired immunosuppressive capacity in EAH, the levels of related mRNA transcripts were examined by RT-qPCR in both NCs and EAH model mice. As a core transcription factor in immunosuppressive regulation, FOXP3 showed markedly decreased expression in Tregs from EAH model mice compared with those from NCs (Figure 3B). Additionally, similar patterns of expression for cytotoxic T-lymphocyte antigen (CTLA-4) and CD103 were discovered in EAH. In the model group, the TIGIT levels were slightly decreased, but the difference was not significant (Figure 3C). Interestingly, we found that the levels of CD39 and CD73, which are known to produce the inhibitory molecule adenosine, were decreased in EAH-derived Tregs (Figure 3D). qPCR analysis further compared cytokine production between the two groups. Tregs from EAH model mice produced lower levels of anti-inflammatory factors (e.g., TGF-β and IL-35) than those from NCs (Figure 3E). Taken together, these data suggest that in EAH, Tregs have impaired immunosuppressive function, as reflected by the downregulated mRNA levels of FOXP3, CD39, CD73, CTLA-4, CD103 and anti-inflammatory cytokines.

### Tregs Have Augmented Glycolysis and Reduced OXPHOS in EAH

**FIGURE 4:**
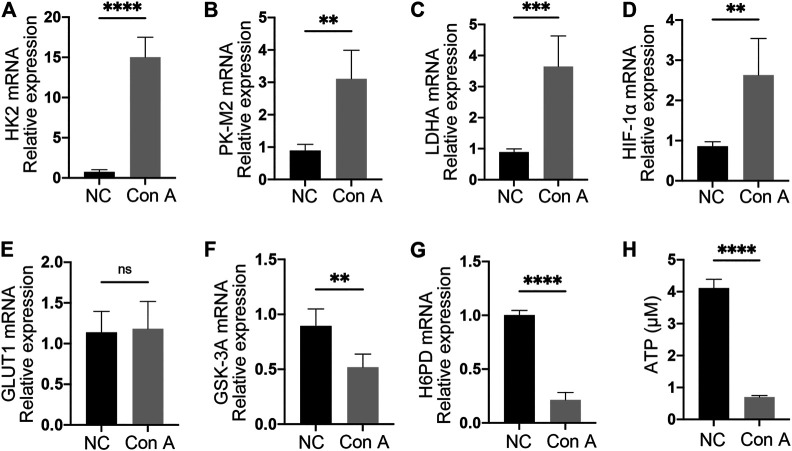
EAH-derived Tregs had enhanced glycolysis and decreased OXPHOS. **(A–C)** The expression of HK2, PK-M2, and LDHA in freshly isolated Tregs from NCs and EAH model mice was assessed by RT-qPCR. **(D–E)** HIF-1α and GLUT1 mRNA levels. **(F–G)** GSK-3A and H6PD mRNA levels. **(H)** Magnetically sorted Tregs from each group were cultured for 24 h. Cells were harvested and lysed to detect the ATP concentration. Gene expression was normalized to that of β-actin.

To investigate alterations in Treg metabolism, Tregs from NCs and EAH model mice were isolated to determine the expression of mRNA transcripts related to glycolysis and OXPHOS and the ATP concentration. The expression of hexokinase 2 (HK2), which serves as a key enzyme in glycolysis, was strikingly upregulated in Tregs from EAH model mice (Figure 4A). The expression of pyruvate kinase-M2 (PK-M2) followed the same increasing trend (Figure 4B). Lactate dehydrogenase A (LDHA) mRNA levels were also significantly elevated in the EAH group (Figure 4C). As an essential stimulator of glycolysis, hypoxia-induced factor 1-α (HIF-1α) showed a considerable rise in its expression level in the EAH group (Figure 4D). Nevertheless, the expression of glucose transporter 1 (GLUT1), which is responsible for glucose uptake, was unchanged (Figure 4E). Cellular ATP levels were markedly decreased in Tregs isolated from EAH model mice, as determined with a luminometer, indicating OXPHOS downregulation (Figure 4F). Additionally, there was a decline in the glycogen synthase kinase 3A (GSK-3A) level of Tregs from EAH model mice, suggesting enhanced glycogenesis (Figure 4G). It was observed that Tregs from EAH model mice had lower levels of hexose-6-phosphate dehydrogenase (H6PD), reflecting an impaired pentose phosphate pathway (Figure 4H). In general, the data demonstrated enhanced glycolysis and impaired OXPHOS in Tregs from EAH model mice.

## Discussion

**FIGURE 5:**
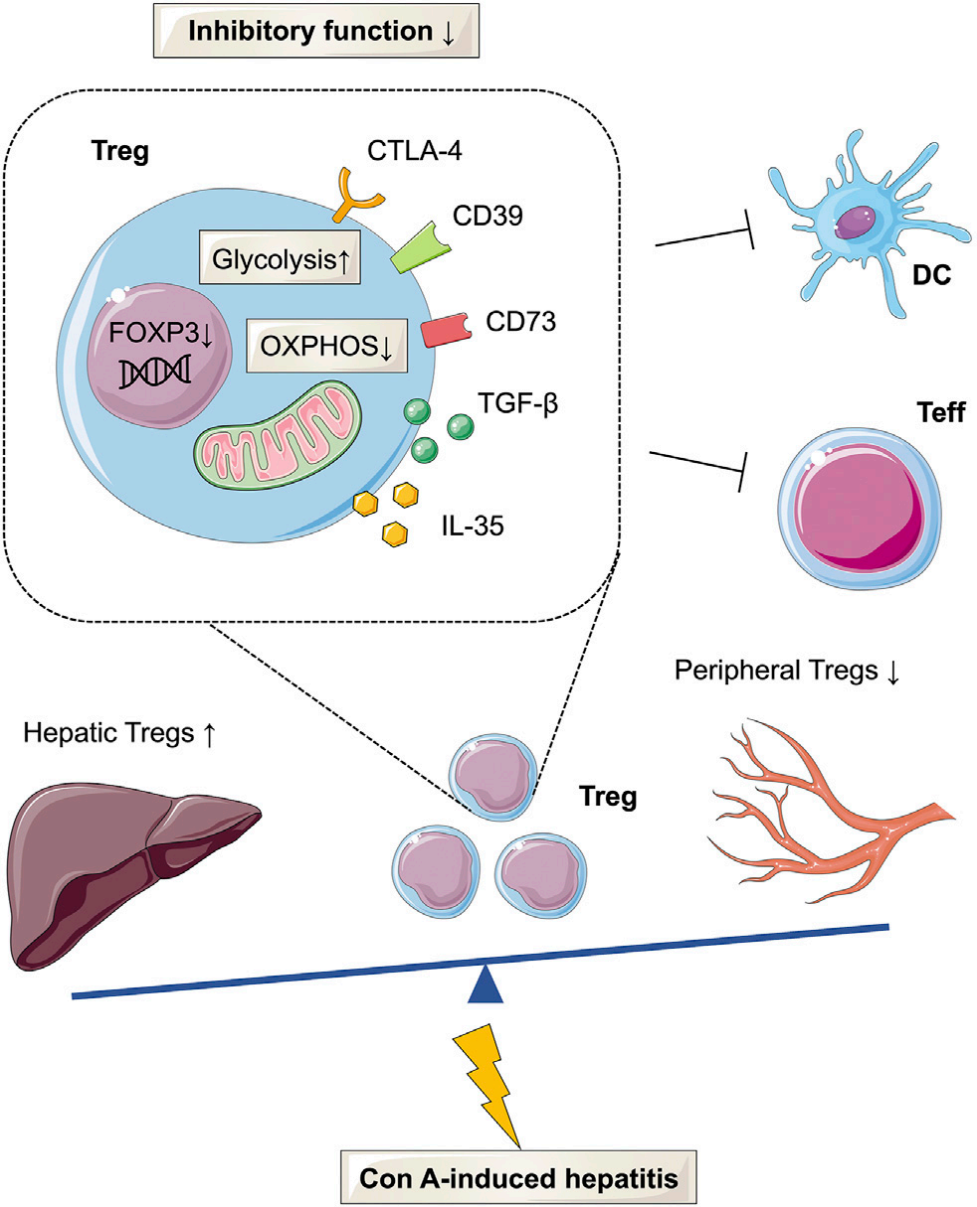
Glucose metabolism reprogramming of Tregs in Con A-induced hepatitis. The frequency of Tregs was decreased in the peripheral but increased in the liver tissues of AIH patients and mouse models. In Con A-induced immune hepatitis, Tregs are functionally impaired and show enhanced glycolysis and decreased OXPHOS.

As important components of the adaptive immune system, Tregs have been reported to modulate the immune microenvironment in the liver (Wang et al., 2020). The present study demonstrated that defective Tregs could promote the progression of Con A-induced immune hepatitis, likely due to increased glycolysis and reduced OXPHOS (Figure 5).

Tregs are a specific subset of CD4+ T cells with coexpression of the IL-2 receptor α-chain (Qiu et al., 2020). The transcription factor FOXP3 is also a characteristic marker of Tregs and is required for Treg development and suppressive functions (Kitagawa and Sakaguchi, 2017). Several studies have reported that the Treg frequency is decreased in the peripheral blood of AIH patients (Liberal et al., 2015; Longhi et al., 2021). However, Peiseler et al. found that circulating Tregs, defined as CD4+ CD25^high^ CD127^low^ FOXP3+ cells, were not reduced in AIH patients compared with HCs (Peiseler et al., 2012). Therefore, we gated CD4+ CD25+ FOXP3+ cells by FCM and performed IHC staining to evaluate the Treg frequency. The findings revealed that Tregs were decreased in the peripheral blood but increased in liver sections in newly diagnosed AIH patients compared with HCs. This phenomenon was further confirmed in a Con A-induced immune hepatitis model. The data are consistent with most studies and indicate that reduced circulating Treg levels and abnormal Treg accumulation in liver tissues may be associated with the etiology of AIH.

Prednisolone plus azathioprine is considered the first-line therapy for AIH (Lohse et al., 2020; Mack et al., 2020). Liberal et al. revealed that patients in remission had a higher percentage of CD4+ CD25+ CD127- Tregs than active AIH patients (Rincon et al., 2018). However, Peiseler et al. found that the frequency of CD25+ CD127- FOXP3+ Tregs was lower in patients in remission than in untreated patients with active AIH (Peiseler et al., 2012). In addition, multicolor immunofluorescence staining of liver biopsies showed that AIH therapy could reduce Treg accumulation in portal areas (Taubert et al., 2014). Similarly, intrahepatic Tregs were also observed to decline during therapy in pediatric AIH (Diestelhorst et al., 2017). To validate whether immunosuppressive treatment can rescue the number of Tregs, were compared the circulating Treg frequency in different groups, including untreated patients, treated patients (3 weeks and 6 months), and patients in biochemical remission, by FCM. The findings indicated that immunosuppressive treatment could reverse the downregulation of circulating Tregs in AIH.

Tregs play essential roles in maintaining immune homeostasis and preventing autoimmunity by suppressing immune responses. Impaired Treg functions have been reported to contribute to various autoimmune diseases (Dominguez-Villar and Hafler, 2018; Mohr et al., 2019). Tregs mainly exert their suppressive effect *via* the following mechanisms (Longhi et al., 2021): 1) Tregs can secrete anti-inflammatory cytokines, such as TGF-β, IL-10, and IL-35; 2) Tregs can hydrolyze proinflammatory ATP to generate immunosuppressive adenosine via CD39 and CD73; 3) Tregs can induce Teff apoptosis via direct contact; and 4) CTLA-4 on Tregs can bind to CD80/CD86 on antigen-presenting cells (APCs), thus downregulating the expression of costimulatory molecules on APCs. Therefore, Tregs can limit the initiation of the immune response by modulating APCs. In the current study, we found that Tregs from EAH model mice had lower mRNA levels of FOXP3, CTLA-4, CD103, TIGIT, CD39, and CD73 than those from NCs. Tregs derived from the Con A-induced hepatitis model also produced less TGF-β and IL-35. These data indicated that Treg dysfunction may be associated with AIH development.

Considering the importance of cellular metabolism to Treg stability and functions, we next examined the shift in glucose metabolism between Tregs from EAH and those from NCs (Kempkes et al., 2019). HK2, PK-M2 and LDHA function as key enzymes in the modulation of glycolysis (Feng et al., 2020). Teffs are known to utilize glycolysis for survival and effector functions (Almeida et al., 2021). In contrast, Tregs mainly depend on OXPHOS to provide the energy supply needed for stability and inhibitory functions (Kurniawan et al., 2020). Enhanced glycolysis has been reported to correlate with functional impairment of Tregs. Previous work indicated that FOXP3 can alter the patterns of glucose metabolism in Tregs, leading to increased glycolysis and decreased OXPHOS (Angelin et al., 2017). Another study identified HIF-1α as a metabolic switch in Tregs that controls the balance between glycolysis and OXPHOS, resulting in a waning of immunosuppression (Rincon et al., 2018). However, the metabolic alterations in Tregs in AIH remain obscure. In the present study, we confirmed that functionally impaired Tregs from EAH model mice had higher levels of HIF-1α and key glycolytic enzymes. Additionally, EAH mouse derived Tregs generated less ATP than those from NCs. This study demonstrated that glucose metabolism reprogramming may promote Treg dysfunction in Con A-induced immune hepatitis. This is the first study to reveal alerted glucose metabolism in Tregs in Con A-induced hepatitis. Adoptive transfer of metabolically engineered Tregs may provide novel insights into AIH treatment.

## Conclusion

In summary, the current study indicates that Tregs are reduced in the peripheral blood but increased in the liver of AIH patients. The same trends were also observed in Con A-induced hepatitis. Furthermore, EAH mouse-derived Tregs exhibited attenuated suppressive functions, enhanced glycolysis and decreased OXPHOS compared with those from NCs.

Glucose metabolism reprogramming in Tregs may impair their suppressive capability, thus leading to immune disorders in Con A-induced immune hepatitis. Therefore, targeting glucose metabolism in Tregs could be a potential approach for AIH treatment.

## Data Availability

The original contributions presented in the study are included in the article/Supplementary Material, further inquiries can be directed to the corresponding author.
