# Supplementary material for: Glucose Metabolism Reprogramming of Regulatory T Cells in Concanavalin A-Induced Hepatitis
Source: Front Pharmacol. 2021 Aug 31;12:726128. doi: 10.3389/fphar.2021.726128 (PMC8438122; doi:10.3389/fphar.2021.726128)
Supplement: Supplementary file 1 [file Table1.DOCX]

Supplementary Material

Supplementary Table 1: Primers used in RT-qPCR analysis.

| **Gene** | **Forward Primer** | **Reverse Primer** |
| --- | --- | --- |
| *Foxp3* | GAACCACGGGCACTATCACA | TTGCTTGAGGCTGCGTATGA |
| *Ctla4* | GACCCAACCTTCAGTGGTGT | CCGGACCTCATCAGTGTTGT |
| *Cd103* | CCTTCTGCATCAAGACCCCA | GGTTCCCGACCAATGTCACT |
| *Tigit* | AGCTGAAGTGACCCAAGTCG | AAGACTGAAGCGACATGCCA |
| *Cd39* | TGCTTACCCGGGACAGTCTA | AGGCCATGAGAGTTGTGAGC |
| *Cd73* | ACGTGCTGTTTTTGGATGCC | AGTGCCATAGCATCGTAGCC |
| *Tgfb1* | CCACCTGCAAGACCATCGAC | CTGGCGAGCCTTAGTTTGGAC |
| *Il35b* | CTCTCAAGTACCGACTCCGC | GTGAGGTCCTGAGCTGACAC |
| *Actin* | GTGACGTTGACATCCGTAAAGA | GCCGGACTCATCGTACTCC |
| *Hk2* | ATGATCGCCTGCTTATTCACG | CGCCTAGAAATCTCCAGAAGGG |
| *Pkm* | CGCCTGGACATTGACTCTG | GAAATTCAGCCGAGCCACATT |
| *Ldha* | CAAAGACTACTGTGTAACTGCGA | TGGACTGTACTTGACAATGTTGG |
| *Hif1a* | GATGACGGCGACATGGTTTAC | CTCACTGGGCCATTTCTGTGT |
| *Glut1* | GCAGTTCGGCTATAACACTGG | GCGGTGGTTCCATGTTTGATTG |
| *Gsk3a* | GCGTTCCCAAGAAGTGGCTTA | GGTCCAGCTTACGCATAATCTG |
| *H6pd* | AAGATGCTCCTAGCGGCAATG | TCCAGGTATAGCTGAAACAGTCC |
